# Robotic-assisted surgery for left-sided colon and rectal resections is associated with reduction in the postoperative surgical stress response and improved short-term outcomes: a cohort study

**DOI:** 10.1007/s00464-024-10749-3

**Published:** 2024-03-18

**Authors:** Abigail R. Ingham, Chia Yew Kong, Tin-Ning Wong, Stephen T. McSorley, Donald C. McMillan, Gary A. Nicholson, Ahmed Alani, David Mansouri, David Chong, Graham J. MacKay, Campbell S. D. Roxburgh

**Affiliations:** 1 Academic Unit of Surgery and School of Cancer Sciences, College of Medicine, Veterinary and Life Sciences, University of Glasgow, New Lister Building, Glasgow Royal Infirmary, Glasgow, G31 2ER UK; 2 Department of General Surgery, Queen Elizabeth University Hospital, 1345 Govan Road, Glasgow, G51 4TF UK; 3 Lister Department of Surgery, Glasgow Royal Infirmary, 84 Castle Street, Glasgow, G4 0SF UK; 4 Academic Unit of Surgery, School of Cancer Sciences, Room 2.60, Level 2 New Lister Building, Glasgow Royal Infirmary, Glasgow, G31 2ER UK

**Keywords:** Robotic surgery, LS surgery, Minimally invasive surgery, Rectal cancer, Colon cancer, Inflammation, Complications, Outcomes, C-reactive protein

## Abstract

**Introduction:**

There is growing evidence that the use of robotic-assisted surgery (RAS) in colorectal cancer resections is associated with improved short-term outcomes when compared to laparoscopic surgery (LS) or open surgery (OS), possibly through a reduced systemic inflammatory response (SIR). Serum C-reactive protein (CRP) is a sensitive SIR biomarker and its utility in the early identification of post-operative complications has been validated in a variety of surgical procedures. There remains a paucity of studies characterising post-operative SIR in RAS.

**Methods:**

Retrospective study of a prospectively collected database of consecutive patients undergoing OS, LS and RAS for left-sided and rectal cancer in a single high-volume unit. Patient and disease characteristics, post-operative CRP levels, and clinical outcomes were reviewed, and their relationships explored within binary logistic regression and propensity scores matched models.

**Results:**

A total of 1031 patients were included (483 OS, 376 LS, and 172 RAS). RAS and LS were associated with lower CRP levels across the first 4 post-operative days (*p* < 0.001) as well as reduced complications and length of stay compared to OS in unadjusted analyses.

In binary logistic regression models, RAS was independently associated with lower CRP levels at Day 3 post-operatively (OR 0.35, 95% CI 0.21–0.59, *p* < 0.001) and a reduction in the rate of all complications (OR 0.39, 95% CI 0.26–0.56, *p* < 0.001) and major complications (OR 0.5, 95% CI 0.26–0.95, *p* = 0.036).

Within a propensity scores matched model comparing LS versus RAS specifically, RAS was associated with lower post-operative CRP levels in the first two post-operative days, a lower proportion of patients with a CRP ≥ 150 mg/L at Day 3 (20.9% versus 30.5%, *p* = 0.036) and a lower rate of all complications (34.7% versus 46.7%, *p* = 0.033).

**Conclusions:**

The present observational study shows that an RAS approach was associated with lower postoperative SIR, and a better postoperative complications profile.

**Supplementary Information:**

The online version contains supplementary material available at 10.1007/s00464-024-10749-3.

The use of robotic-assisted surgery (RAS) for colorectal cancer resections has become more prevalent globally in the past decade. The growth has primarily been driven by surgeon and patient preference with perceived improvements in short-term outcomes compared to laparoscopic surgery (LS) or open surgery (OS). When compared to LS surgery, a number of studies report reduced rates of conversion for colonic and rectal resections [1–5]. One large scale randomised controlled study, the ROLARR trial [4], conducted in the early years of colorectal RAS, recruited patients between 2011 and 2014 comparing robotic and LS for rectal cancer resections. The primary outcome was conversion rate. The conversion rate in the robotic arm was 8.1%, one of the lowest rates obtained in a trial of minimally invasive rectal cancer surgery. However, the trial did not meet its primary endpoint due to a lower than anticipated rate of conversion in the LS arm (12.2%) and, as a result, a statistically significant difference was not achieved. Of interest, no significant differences in complication rates were observed up to 6 months postoperatively. Multiple studies since have reported lower rates of complications with colorectal RAS as well auced length of stay (LOS) and improved bladder/ sexual function [6–10]. In the face of this contrasting evidence, the growth of colorectal RAS has continued and according to Intuitive Surgical, general/ colorectal RAS is now the highest volume RAS user globally surpassing urology and gynaecological surgery in recent years. In the UK, urology remains the highest volume speciality [10, 11].

Serum acute phase reactants including C-reactive protein (CRP) are sensitive measures of the perioperative systemic inflammatory response (SIR). Multiple studies support serial CRP monitoring after colorectal surgery for early identification of infective complications. In particular, a post-operative day 3 CRP ≥ 150mg/l suggests increased risk of infective complications [12–14] such as intra-abdominal collection or anastomotic leak. Conversely, lower CRP levels on day 2 and day 3 confer a high negative predictive value for development of infective complications and provide reassurance to facilitate early discharge from hospital [15].

It is well-established conventional LS reduces the SIR insult in the perioperative period [13, 17]. In addition to operative approach, many other clinical and pathological factors contribute to the magnitude of the postoperative stress response; including obesity, tumour stage and presence of a preoperative SIR [16, 17]. The impact of robotic-assisted surgery on serum inflammatory profiles compared with LS and OS has not been investigated in detail.

In 2021, following a National Planning process, the Scottish Government made a significant investment in robotic-assisted surgery in Scotland to support development of RAS in key specialities (colorectal surgery, urology, thoracic surgery, head and neck surgery and gynaecologic oncology) with the aim of reducing rates of OS and improving short-term outcomes across key cancer types. In Glasgow, 2 da Vinci Xi systems were sited at 2 large teaching hospitals, the Glasgow Royal Infirmary (GRI) and the Queen Elizabeth University Hospital (QEUH) to support colorectal RAS in NHS Greater Glasgow and Clyde. Following implementation of these systems, all resectable left-sided colonic and rectal cancers were directed towards robotic trained surgeons. This present study reports the short-term outcomes from two years of full implementation at GRI. As the transition to RAS for left-sided/ rectal cancer resections was immediate, as of May 2021, all such cases are now managed with RAS. Furthermore, outcomes can be directly compared for patients with these tumours compared with a retrospective cohort of left sided/ rectal resections managed with LS or OS in the previous 14 years at GRI (2008–2022). Specifically, the aim is to compare short-term outcomes including complication rate and LOS but also quantification of the SIR following OS, LS and RAS for left-sided and rectal cancer operations.

## Methods

### Patients and methods

Since 2008, all patients undergoing colorectal cancer surgery at GRI have been entered into a prospectively maintained departmental database. Since the implementation of colorectal RAS in Glasgow in May 2021, all robotic surgical resections at GRI have been entered into a prospective database for audit and research purposes. Data from patients who have undergone resection for left-sided (high anterior resections for descending and sigmoid colon cancer) and rectal (low anterior resection or abdominoperineal resection) cancers have been included in this study. Benign pathology has been excluded.

Data were retrieved using electronic hospital records and added to a prospectively maintained, pseudo-anonymised database which included patient demographics and clinico-pathological characteristics such as surgical approach, operative details, perioperative complications up to 30 days graded according to the Clavien-Dindo (CD) classification [17], postoperative blood results, postoperative imaging assessment, blood transfusion rates and LOS.

Operative approach was categorised into OS, LS, and RAS and is the main explanatory variable of interest in this study. When a substantial component of a planned hybrid procedure was performed via an OS approach (e.g., planned OS total mesorectal excision following a LS splenic flexure mobilisation), this was considered an OS operation. Conversions were noted but remained in their original operative approach cohort as intention to treat. In the unmatched cohort, where analyses focus on the comparing postoperative inflammatory response profiles according to surgical approach, we specifically excluded converted cases.

Operation types were coded into the following categories: high anterior resection (HAR), low anterior resection (LAR) with or without primary anastomosis, abdomino-perineal resection (APR), and other procedures including subtotal colectomy and panproctocolectomy.

HARs were defined by an anastomosis at or above the peritoneal reflection, where the underlying pathology included distal descending, sigmoid, rectosigmoid, and upper rectal tumours (12-15cm from anorectal ring). LARs were defined as an anastomosis below the peritoneal reflection and included rectal tumours within 12 cm from anorectal ring as per preoperative MRI and colonoscopy. For most patients who undergo a low anterior resection, our practice is to perform a defunctioning loop ileostomy to protect the low pelvic anastomosis. APRs involved a perineal excision due to tumour involvement of the levator muscles, sphincter muscles or anal canal and in some cases involved plastic surgical reconstruction. Multi-visceral resections including beyond TME or pelvic exenterative surgery were excluded.

Tumours were staged according to TNM classification as per Royal College of Pathologists Guidelines [18].

Patients with low volume oligo-metastatic disease (M1) were included within the study as all patients have been selected for surgical intervention with curative intent. Those with widespread metastatic disease or those selected for palliative procedures were excluded.

Where neoadjuvant therapy was administered for locally advanced rectal cancer, based on multidisciplinary team recommendation, this was most commonly in the form of long course chemo-radiation delivered over 5 weeks (45–54 Gy in 25 fractions) with concomitant fluoropyrimidine-based chemotherapy regimens [19].

The preoperative and postoperative systemic inflammatory responses were measured using serum C-reactive protein (CRP) levels (mg/L). Preoperative CRP was collected 1–28 days prior to their operation as part of their routine preoperative assessment. Serum concentrations of CRP (mg/l) were measured for all patients using an auto-analyser (Architect; Abbot Diagnostics, Maidenhead, UK) with a lower detectable limit of 0.2 mg/L. There was no change in thresholds or measurement methods throughout the study period. Patients were grouped in line with previously published thresholds. A preoperative CRP ≥ 10mg/L has previously been related to risk of post-operative complications and disease outcome [20–23, 26]. Normal CRP values are defined as 0-9mg/L within our lab.

It is routine practice in our institutions to measure CRP following colorectal cancer resection on postoperative days 1–4 or until discharge from hospital. The postoperative CRP threshold used to group cohorts was based on previous studies (**≥ **150 mg/L) [12–14].

Where radiological assessments (CT scanning) were requested in the postoperative period to assess for potential complications, this data were captured.

Complication rate was classified using the validated Clavien-Dindo classification [17] with a range from 0 (no complication) to 5 (mortality) based on the degree of intervention required to treat the complication. Major complications were defined as complications ranging from CD grade 3–5, whereas the development of any complications was defined as developed complications of any CD grade (Grade 1–5).

Anastomotic leaks were identified from postoperative CT imaging and severity graded according to the impact on clinical management as per the International Study Group of Rectal Cancer grading system; A- results in no change in management, B- requires active therapeutic intervention short of a laparotomy, C- requires relaparotomy [24].

This study was reported in accordance and complies with the accordance with the Strengthening the Reporting of Observational Studies in Epidemiology (STROBE) guidelines [25].

### Statistical analysis

Clinical and pathological data were grouped according to standard thresholds. Categorical data were reported as patient numbers and relative proportions within the individual OS, LS and RAS groups. Differences in categorical variables were compared using the *χ*2 test or Fisher’s exact test for trend. Continuous variables were presented using median values with interquartile ranges. Differences in continuous variables between OS, LS and RAS were compared using the Kruskal–Wallis test. Differences in continuous variables between LS and RAS were compared with Mann–Whitney U tests. A *P* value of < 0.05 was considered statistically significant.

Univariate binary logistic regression models were explored between key preoperative clinical variables and key outcomes of interest including CRP levels exceeding 150 mg/L at post-operative day (POD) 3, the development of any complications and the development of major complications. Significant preoperative variables which were statistically significant (*p* value < 0.05) on univariate analyses were then included in the backward conditional multivariate binary logistic regression model if more than one of these preclinical variables were statistically significant. Odds ratios were reported with 95% confidence intervals.

Finally, within the whole group, a propensity scores matched analysis comparing patients undergoing LS and those undergoing RAS was performed. LS and RAS patients were matched in a 1:1 ratio. Propensity scores were calculated from selected variables (age, sex, BMI, ASA, T-stage, N-stage, type of procedure and preoperative CRP level). Patients were matched by the closest propensity score on the logit scale with a calliper width < 0.05, with a randomised order of matched selection, and without replacement. The appropriateness of the propensity score matching was assessed visually by the frequency of propensity scores assigned to each group before and after matching. The matched pairs’ categorical and continuous outcome variables were compared between the matched with McNemar and Wilcoxon signed-rank tests, respectively.

All statistical analyses were performed using SPSS Version 29 (IBM, Armonk, Ny, USA).

## Results

### Patient demographics

**Fig. 1:**
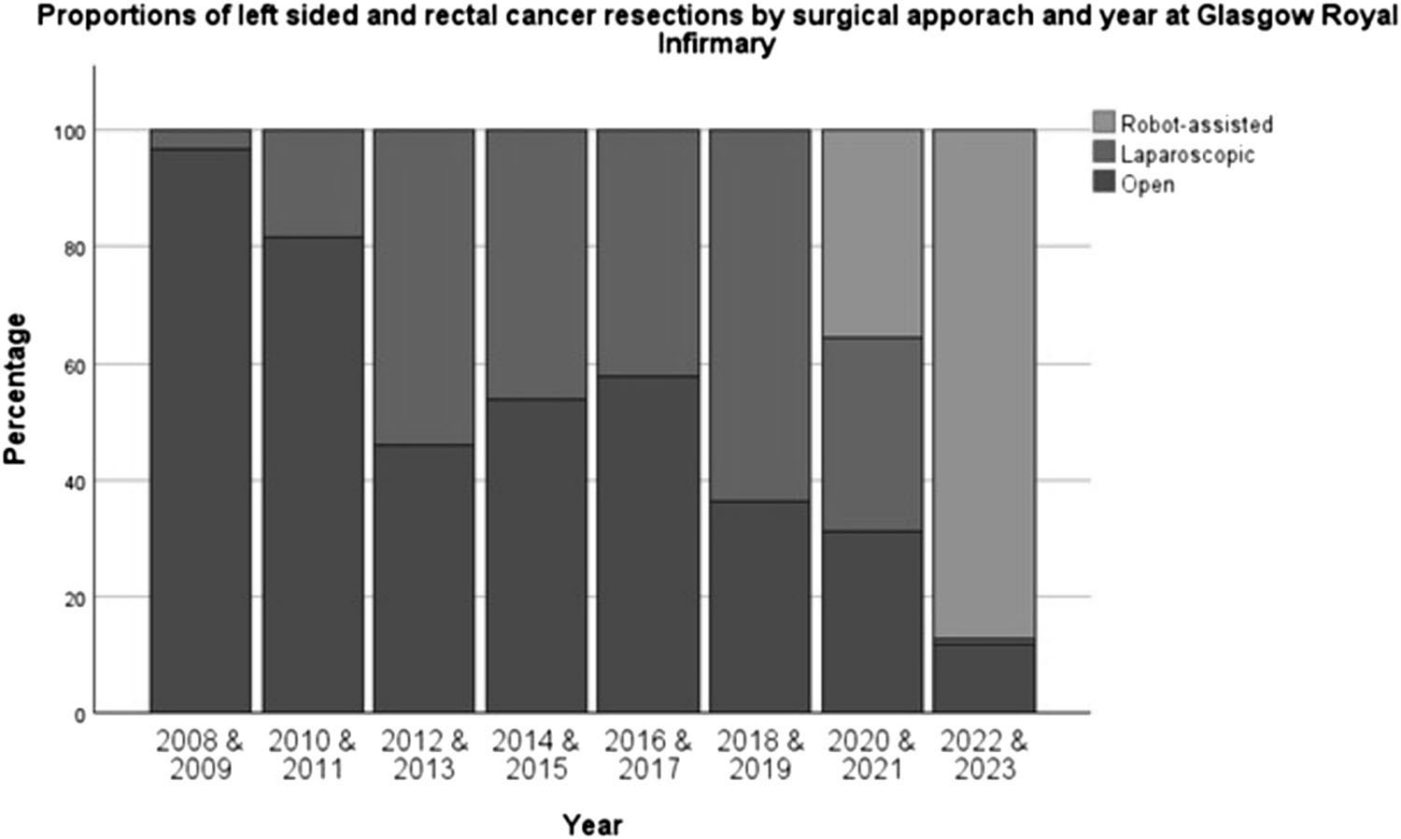
Surgical approach for left-sided and rectal cancer resections by year at GRI from 2008 to 2023. (*N* = 1031)

Between January 2008 and May 2021, 830 patients underwent surgery for left-sided and rectal cancer at GRI (462 OS, 368 LS). Since the introduction of RAS in May 2021 to March 2023, 201 patients underwent surgery for left-sided and rectal cancer (21 OS, 8 LS and 172 RAS) at GRI. Figure 1 and Table 1 shows the operative approach for these operations over this time-period. Rates of minimally invasive surgery (MIS) were highest in 2022/2023 when over 85% of left-sided colon and rectal cancer patients were managed with MIS. RAS commenced at GRI in May 2021 with 2 surgeons commencing simultaneously (CR and GM) followed by DC (September 21) and DM (April 22). Initial cases selected for each surgeon were deemed to be technically straightforward high anterior resections but within 2 months all left-sided and rectal resections were directed to the RAS surgical team (Fig. 1).

**Table 1:** Baseline clinical, pathological characteristics and clinical outcomes of all patients (*n* = 1031) who had a resection performed for left-sided and rectal cancer based on operative approach (Chi squared test for trend for categorical data

All patients	OS	LS	RAS	*P* value* (OS vs LS vs RAS)	*P* value** (LS vs RAS)
Total resections *n* = 1031	*N* = 483 (%)	*N* = 376	*N* = 172 (%)		
High	196 (40.6%)	178 (47.3%)	73 (42.4%)	< 0.001	0.071
Low	126 (26.1%)	149 (39.6%)	62 (36%)		
APR	113 (23.4%)	28 (7.4%)	24 (14%)		
Other	48 (9.9%)	21 (5.6%)	13 (7.6%)		
Year 2008–2013	199 (41.3%)	88 (23.5%)	0	< 0.001	< 0.001
Year 2013–2018	180 (37.3%)	176 (46.9%)	0		
Year 2019–2023	103 (21.4%)	111 (29.6%)	172 (100%)		
Age ≤ 54	78 (16.1%)	77 (20.5%)	35 (20.3%)	0.304	
Age 55–74	291 (60.2%)	225 (59.8%)	105 (61%)		
Age ≥ 75	114 (23.6%)	74 (19.1%)	32 (18.6%)		0.951
Sex Female	214 (44.3%)	143 (38%)	74 (43%)	0.170	0.268
BMI < 18.5 kg/m2	16 (3.3%)	3 (0.8%)	4 (2.3%)	0.004	0.052
BMI 18.5 – 24.9 kg/m2	141 (29.3%)	94 (25%)	29 (16.9%)		
BMI 25- 29.9 kg/m2	170 (35.3%)	146 (38.8%)	81 (47.1%)		
BMI ≥ 30 kg/m2	154 (32%)	133 (35.4%)	58 (33.7%)		
ASA ≥ 3	185 (38.5%)	98 (26.1%)	62 (36%)	< 0.001	0.018
T1	50 (10.4%)	75 (19.9%)	29 (17.2%)	< 0.001	0.696
T2	72 (15%)	85 (22.6%)	34 (20.1%)		
T3	251 (52.2%)	184 (48.9%)	89 (52.7%)		
T4	108 (22.5%)	32 (8.5%)	17 (10.1%)		
N0	289 (60%)	251 (66.8%)	114 (66.3%)	0.112	0.739
N1	127 (26.3%)	93 (24.7%)	40 (23.3%)		
N2	66 (13.7%)	32 (8.5%)	18 (10.5%)		
M0	467 (96.7%)	368 (97.9%)	166 (96.5%)	0.523	0.349
M1	16 (3.3%)	8 (2.1%)	6 (3.5%)		
Preop CRP ≥ 10 mg/L	31.6%	14.6%	17.5%	< 0.001	0.385
Median POD1 CRP (mg/L)	107 (76–143)	58 (39–85)	45 (31–69)	< 0.001	< 0.001
Median POD2 CRP (mg/L)	177 (127–235)	102 (62–150)	80 (50–123)	< 0.001	0.001
Median POD3 CRP (mg/L)	162 (107–231)	102 (63–169)	86 (53–148)	< 0.001	0.037
Median POD4 CRP (mg/L)	126 (75–195)	81 (46–150)	67 (36–67)	< 0.001	0.066
POD2 CRP ≥ 150 mg/L	62.2%	24.9%	16%	< 0.001	0.026
POD3 CRP ≥ 150 mg/L	54.1%	28.4%	21.3%	< 0.001	0.09
Median length of inpatient stay	12 (8–17)	6 (4–10)	6 (4–9)	< 0.001	0.057
Any complications within 30 days	289 (60%)	166 (44.1%)	61 (35.5%)	< 0.001	0.055
Major complications CD3-5 within 30 days	68 (14.1%)	37 (9.8%)	12 (7%)	0.021	0.276
Death within 30 days	9 (1.9%)	3 (0.9%)	0	0.066	0.219
Post-operative CT imaging within 30 days	161 (33.4%)	93 (25%)	37 (21.6%)	0.003	0.394
Post-operative transfusion within 30 days	86 (18%)	13 (3.6%)	7 (4.1%)	< 0.001	0.767
Post-operative SSI within 30 days	91 (19.9%)	30 (8.6%)	14 (8.1%)	< 0.001	0.867
Readmission to hospital within 30 days	51 (10.6%)	33 (8.9%)	13 (7.6%)	0.624	0.629
Re-operation within 30 days	41 (8.5%)	29 (7.8%)	8 (4.7%)	0.428	0.173
Anastomotic leak rate within 30 days	6.4%	5.5%	3.4%	0.388	0.039
Conversion to open	NA	34 (9.1%)	7 (4.1%)	NA	0.039

Patients undergoing conversions have been removed from analyses of CRP outcomes data

*Kruskal–Wallis test for continuous data between OS, LS and RAS and

**Mann Whitney U test for continuous data between LS and RAS)

**Fig. 2:**
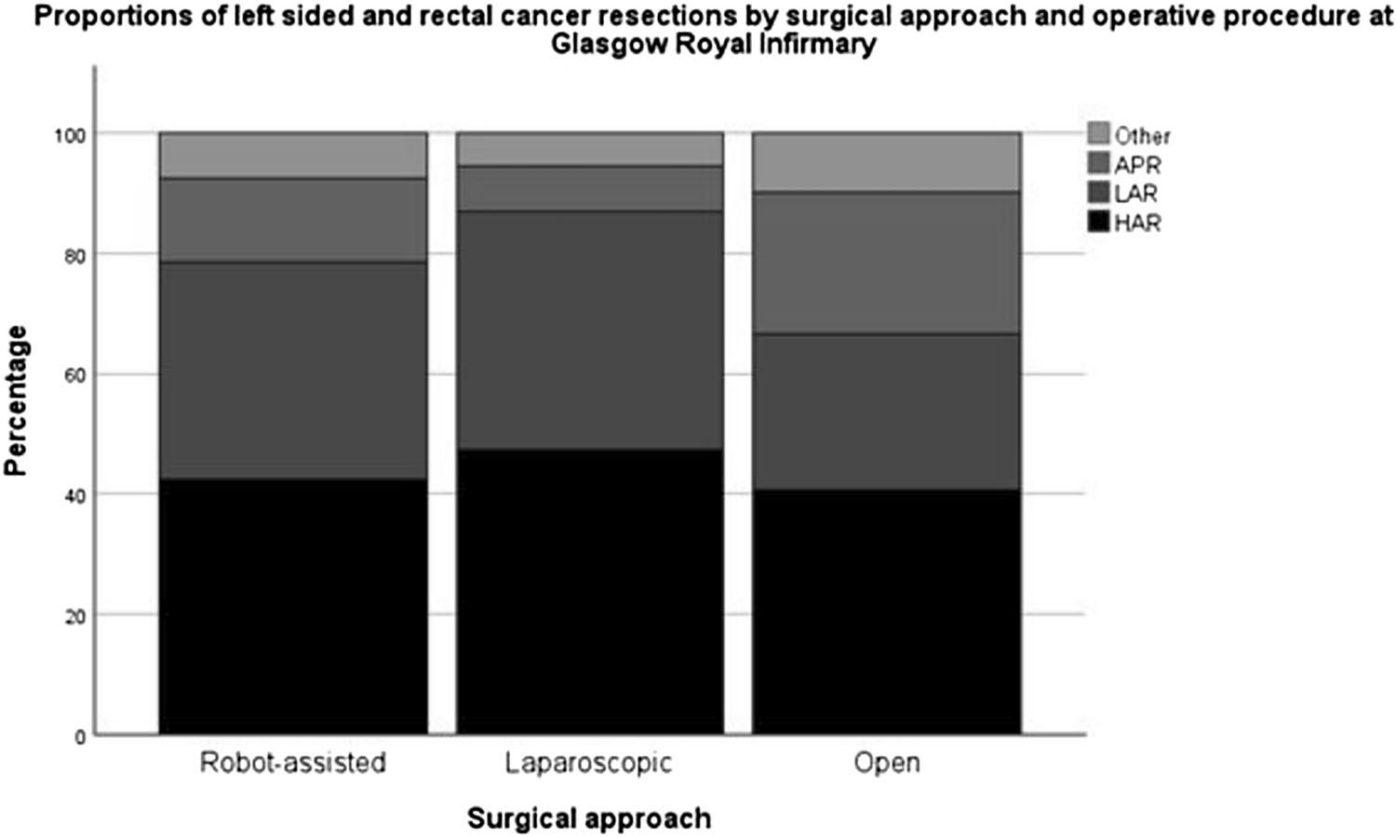
Operative distribution of all left-sided colorectal cancer resections by surgical approach. (*N* = 1031)

Baseline characteristics of each group in the whole unmatched cohort stratified by surgical approach is provided in Table 1. Comparisons for trend are made across all 3 surgical approach cohorts as well as specifically for LS vs RAS approaches. There was no difference in the sex, age, nodal status or metastatic disease status between the 3 cohorts. In each cohort the most common procedure was HAR, followed, respectively, by LAR, APR, and other procedures, which includes subtotal colectomy and panproctocolectomy. In the OS cohort, 40.6% of cases were HAR followed by 26.1% LAR and 23.4% APR. In the LS cohort 47.3% were HARs, 39.6% LARs and 7.4% APRs and in the RAS cohort 42.4% were HARs, 36% were LARs and 14% APRs (Fig. 2). There was a significant trend towards an increasing proportion of overweight and obese patients BMI ≥ 25 kg/m2 in the minimally invasive approaches compared to OS (OS 67.3% LS 74.2% RAS 80.8%, *p* = 0.004).

There was a statistically significant difference in the proportions of these procedures across the 3 operative approaches (*p* < 0.001) but across LS versus RAS patients alone, there were no statistically significant differences (*p* = 0.071). Patients treated with an OS approach had the highest proportion of patients with ASA ≥ 3 (38.5%) followed by RAS and LS approaches (36% and 26.1%, respectively) (*p* < 0.001 for all groups; *p* = 0.018 for RAS versus LS). There is a significant trend towards OS cohort having more advanced T-stage including a higher proportion of T4 disease on pathology than both LS and RAS (p < 0.001) but no difference in nodal status or whether metastatic disease is present. There were no differences in trend for T-stage when comparing the LS and RAS groups specifically.

There was a trend towards a higher proportion of patients with pre-op CRP > 10 mg/l in the OS group (31.6%) (*p* < 0.001) but there were no statistically significant differences between LS and RAS when compared specifically (*p* = 0.385).

OS had the highest rates of neoadjuvant therapy followed by RAS (OS 27.6% vs LS 12% vs RAS 20.5%).

All patients had a minimum follow-up of 90 days.

### Inflammation in the postoperative period (CRP)

**Fig. 3:**
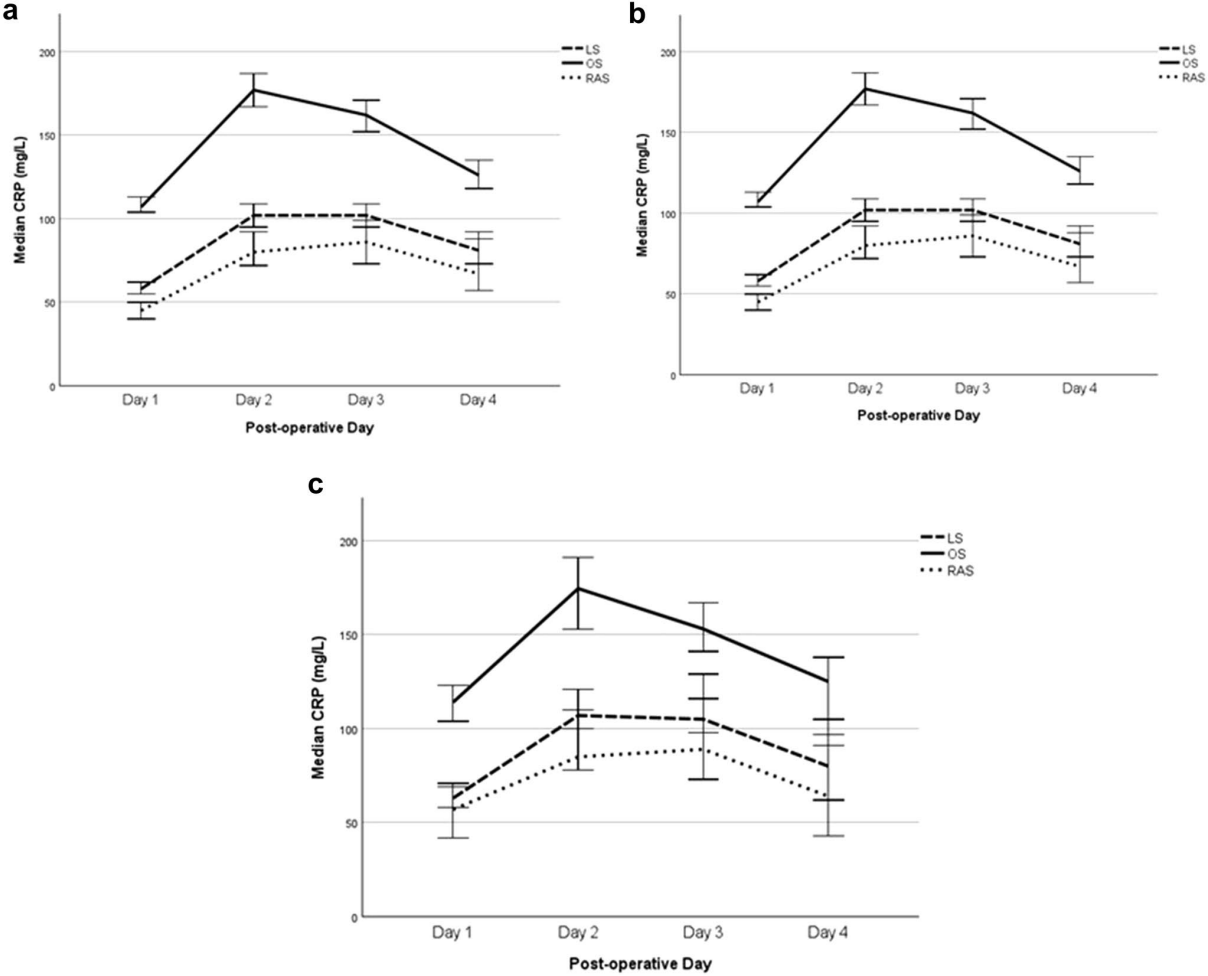
**a** Median daily CRP level trends of all patients undergoing surgery for left-sided or rectal stratified by surgical approach. Patients undergoing conversions have been removed from analyses of CRP outcomes data. **b** Median daily CRP level trends of all patients undergoing high anterior resections stratified by surgical approach. Patients undergoing conversions have been removed from analyses of CRP outcomes data. **c** Median daily CRP level trends of all patients undergoing low anterior resections stratified by surgical approach. Patients undergoing conversions have been removed from analyses of CRP outcomes data

Across all patients there were significant differences in the perioperative inflammatory profiles between the different surgical approach groupings when comparisons for trend were assessed for trends between the OS, LS and RAS approaches (Table 1 and Fig. 3). Comparing for trends between RAS versus both OS and LS, median POD 1, 2, 3, and 4 CRP were significantly lower after RAS, likewise fewer RAS patients had a CRP** ≥ **150 on POD 2 and 3 (all *p* < 0.001).

When comparing RAS to LS specifically, there were significant differences in POD 1, 2 and 3 CRP levels (*p* < 0.001, *p* = 0.001 and *p* = 0.037, respectively). There was a trend towards lower POD 4 CRP levels with an RAS approach which was not statistically significant (*p* = 0.066). There was a lower proportion of RAS patients with a POD 2 CRP exceeding 150 mg/l (*p* = 0.026). There was a trend towards a lower proportion of patients with a POD3 CRP exceeding 150 mg/l with an RAS versus LS approach, but this was not statistically significant (*p* = 0.09).

### Perioperative outcomes

Across all patients undergoing left-sided colon cancer and rectal cancer surgery, there was a trend towards a longer LOS in OS compared to LS and RAS (median of 12 days OS vs 6 days for LS and RAS), *p* < 0.001 (Table 1). There were no statistically significant differences in LOS between RAS and LS specifically.

The rate of patients experiencing any complications within the first 30 post-operative days were most frequent in the OS cohort followed by LS and RAS; [60% OS vs 44.1% LS vs 35.5% RAS, *p* < 0.001). The rate of major complications was significantly lower in the RAS cohort (7%) vs LS (9.8%) and OS (14.1%) (*p* = 0.021). There were no statistically significant differences in the rate of all or major complications between RAS and LS specifically although there was a trend towards a reduced all-complications (CD1-5) rate in RAS versus LS (35.5% versus 44.1%, respectively, (*p* = 0.055)].

No mortality was observed in the RAS cohort vs 0.9% in the LS cohort and 1.9% in the OS cohort. There were no statistically significant differences in mortality between the cohorts in whole group comparisons or between RAS and LS.

In the whole group, there was a trend towards fewer patients requiring a postoperative CT scan in the minimally invasive approaches versus OS to investigate potential complications within 30 days, (OS 33.4% vs LS 25% vs RAS 21.6%, *p* = 0.003). Comparing RAS versus LS specifically, these differences were not statistically significant.

The postoperative blood transfusion rate was lower with minimally invasive surgery approach versus OS approach (OS 18% vs LS 3.6% vs RAS 4.1%, *p* < 0.001). Comparing RAS versus LS specifically, these differences were not statistically significant.

The rate of surgical site infection (SSI) within 30 days was lower in RAS and LS surgery compared to OS (OS 19.9% vs LS 8.6% vs RAS 8.1%, *p* < 0.001). Anastomotic leak rates were numerically lower in RAS when compared to LS and OS but did not reach significance (OS 6.4% (4 × Grade B, 25 × Grade C) vs LS 5.5% (2 × Grade B, 13 × Grade C) vs RAS 3.4% (1 × Grade B, 4 × Grade C 2.7%), p = 0.388). Comparing LS versus RAS specifically, RAS was associated with a significantly lower anastomotic leak rate (*p* = 0.039).

There was a trend in favour of RAS for a reduced rate of reoperation or readmission within 30 days although these were not statistically significant in whole group or RAS versus LS comparisons.

There were fewer conversions in the RAS cohort (4.1%) vs LS (9.1%) (*p* = 0.039).

### Binary logistic regression

Binary logistic regression analysis was performed to determine which preoperative clinical factors including surgical approach determined magnitude of the postoperative inflammatory response and development of any and major postoperative complications, respectively (Tables 4 and5).

The relationship between preoperative clinical characteristics and POD3 CRP ≥ 150 mg/L are shown in Table 2. On univariate binary logistic analysis, male patients (*p* = 0.048), preoperative CRP ≥ 10mg/L (*p* < 0.001), advancing *T* stage (*p* < 0.001), operative procedure type (HAR = reference) (LAR *p* = 0.243; APR *p* < 0.001, Other procedures *p* = 0.007), and higher ASA (*p* = 0.006) were associated with a CRP ≥ 150mg/L at day 3 postoperatively. In addition, LS and RAS approaches (both *p* < 0.001), later year of surgery (2014–2018 *p* = 0.003; 2019–2023 *p* < 0.001) were also associated with lower CRP levels at day 3 post-operatively (both *p* < 0.001). On multivariate analysis, male sex (OR 1.42, 95% CI 1.07–1.89, *p* = 0.016), preop CRP ≥ 10 (OR 2.37, 95% CI 1.70–3.31, *p* < 0.001) and operative procedure type was also associated with higher CRP levels at POD 3. Using HAR as the reference group, all other procedure types were independently associated with elevated POD3 CRP levels (LAR OR 1.58, 95% CI 1.14–2.21, *p* = 0.007; APR OR 2.34, 95% CI 1.56–3.51, *p* < 0.001; Other procedures OR 1.73 95% CI 1.02–2.93, *p* = 0.041). Finally, LS and RAS approaches and later year of surgery were independently associated with lower CRP levels at 3 days postoperatively (LS OR 0.45, 95% CI 0.33–0.62; RAS OR 0.35 95% CI 0.21–0.59 (both *p* < 0.001)) and (2014–2018 OR 0.67, 95% CI 0.47–0.93, *p* = 0.018; 2019–2023 OR 0.54 95% CI 0.36–0.81, *p* = 0.003) (Table 2).

**Table 2:** Binary Logistic regression comparing preoperative factors with a POD 3 CRP ≥ 150 mg/L in the whole group

	POD3 CRP ≥ 150 mg/L			
	Univariate analysis OR (95%CI)	*P* value	Multivariate analysis OR (95%CI)	*P* value
Age				
≤ 54	1.00 (reference)			
55–74	1.22 (0.85–1.67)	0.310		
≥ 75	1.24 (0.83–1.87)	0.296		
Sex				
F	1.00 (reference)		1.00 (reference)	
M	1.0 (0.99–1.67)	0.048	1.42 (1.07–1.89)	0.016
BMI ≥ 30				
< 30 kg/m^2^	1.00 (reference)			
≥ 30 kg/m^2^	1.26 (0.96–1.65)	0.099		
ASA				
1–2	1.00 (reference)			
3–4	1.46 (1.12–1.91)	0.006	-	0.173
Pre-op CRP ≥ 10mg/L				
< 10 mg/L	1.00 (reference)		1.00 (reference)	
≥ 10 mg/L	2.6 (1.92–3.53)	< 0.001	2.37 (1.70–3.31)	< 0.001
T stage				
T1 and T2	1.00 (reference)			
T3 and T4	1.63 (1.24–2.16)	< 0.001	–	0.233
Node involvement				
Negative	1.00 (reference)			
Positive	1.15 (0.88–1.49)	0.315		
TNM Stage				
I–II	1.00 (reference)			
III	1.13 (0.86–1.49)	0.368		
IV	1.6 (0.75–3.41)	0.226		
Surgical approach				
Open	1.00 (reference)		1.00 (reference)	
Laparoscopic	0.34 (0.25–0.46)	< 0.001	0.45 (0.33–0.62)	< 0.001
Robot-assisted	0.23 (0.15–0.35)	< 0.001	0.35 (0.21–0.59)	< 0.001
Year of operation				
2008–2013	1.00 (reference)		1.00 (reference)	
2014–2018	0.62 (0.45- 0.85)	0.003	0.67 (0.47–0.93)	0.018
2019–2023	0.42 (0.30- 0.58)	< 0.001	0.54 (0.36–0.81)	0.003
Type of resection				
HAR	1.00 (reference)			
LAR	1.2 (0.89–1.62)	0.243	1.58 (1.14–2.21)	0.007
APR	2.2 (1.54–3.21)	< 0.001	2.34 (1.56–3.51)	< 0.001
Others	1.94 (1.20–3.12)	0.007	1.73 (1.02–2.93)	0.041

Any conversions to open were excluded from this analysis. (*N* = 990)

The relationship between development of complications and preoperative clinical characteristics is shown in Table 3. On univariate analysis, age ≥ 75 (0.032), male sex (*p* = 0.008), pre-op CRP ≥ 10mg/L (*p* = 0.003), high ASA (*p* < 0.001) and operative procedure type (HAR = reference) (LAR *p* = 0.002; APR *p* < 0.001, Other procedures *p* < 0.001) were associated with increased likelihood of developing any complications. Only an LS and RAS approach were associated with less likelihood of developing any complications both *p* < 0.001. On multivariate analysis, male sex (OR 1.38, 95% CI 1.06–1.80, p = 0.017), ASA 3 and 4 (OR 1.64, 95% CI 1.24–2.17, *p* < 0.001) and pre-op CRP < 10 mg/L (OR 1.38 95% CI 1.00–1.89, *p* = 0.049) were associated with a higher likelihood of developing any complications. Operative procedure type was also associated with the development of any major complications. Using HAR as the reference group, all other procedure types were independently associated with any (CD 1–5) complications (LAR OR 1.88, 95% CI 1.39–2.54, *p* < 0.001; APR OR 3.04, 95% CI 2.04–4.51, *p* < 0.001; Other procedures OR 2.22, 95% CI 1.33–3.71, *p* = 0.002). An LS and RAS approach was associated with a reduced likelihood of developing any complications (OR 0.63, 95% CI 0.47–0.85, *p* = 0.002 and OR 0.39 (0.26–0.56), *p* < 0.001, respectively.

**Table 3:** Binary logistic regression comparing preoperative factors with development of any complications in the whole group

	All complications			
	Univariate analysis OR (95%CI)	*P* value	Multivariate analysis OR (95%CI)	*P* value
Age				
≤54	1.00 (reference)			
55–74	1.19 (0.86–1.64)	0.305	–	0.879
≥ 75	1.53 (1.04–2.27)	0.032	–	0.351
Sex				
F	1.00 (reference)		1.00 (reference)	
M	1.4 (1.1–1.79)	0.008	1.38 (1.06–1.80)	0.017
ASA				
1–2	1.00 (reference)		1.00 (reference)	
3–4	1.67 (1.29–2.17)	< 0.001	1.64 (1.24–2.17)	< 0.001
BMI ≥ 30				
< 30 kg/m^2^	1.00 (reference)			
≥ 30 kg/m^2^	1.19 (0.92–1.55)	0.18		
Pre-op CRP ≥ 10mg/L				
< 10 mg/L	1.00 (reference)			
≥ 10 mg/L	1.58 (1.17–2.12)	0.003	1.38 (1.00–1.89)	0.049
T stage				
T1 and T2	1.00 (reference)			
T3 and T4	1.2 (0.92–1.55)	0.172		
Node involvement				
Negative	1.00 (reference)			
Positive	0.97 (0.75–1.25)	0.809		
TNM Stage				
I–II	1.00 (reference)			
III	0.95 (0.73–1.24)	0.713		
IV	1.5 (0.71–3.16)	0.291		
Surgical approach				
Open	1.00 (reference)		1.00 (reference)	
Laparoscopic	0.53 (0.4–0.69)	< 0.001	0.63 (0.47–0.85)	0.002
Robot-assisted	0.37 (0.26–0.53)	< 0.001	0.39 (0.26–0.56)	< 0.001
Year of operation				
2008–2013	1.00 (reference)			
2014–2018	1.0 (0.74- 1.38)	0.951		
2019–2023	0.98 (0.72- 1.33)	0.885		
Type of resection				
HAR	1.00 (reference)			
LAR	1.58 (1.19–2.10)	0.002	1.88 (1.39–2.54)	< 0.001
APR	3.23 (2.21–4.71)	< 0.001	3.04 (2.04–4.51)	< 0.001
Others	2.60 (1.59-4.23)	< 0.001	2.22 (1.33-3.71)	0.002

The relationship between preoperative clinical characteristics and the development of major complications (CD grade 3 +) are shown in Table 4. On univariate analysis, only advancing T-stage of tumours (*p* = 0.044) were associated with increased likelihood of major complications. Likewise, only an RAS approach (*p* = 0.017) was associated with a reduced likelihood of developing major complications. Similarly, on multivariate analysis, only a RAS approach (OR 0.5 95% CI 0.26–0.95, *p* = 0.036) was independently associated with a reduced likelihood of developing major complications.

**Table 4:** Binary logistic regression comparing preoperative factors with development of major complications in the whole group

	Major complications			
	Univariate analysis OR (95%CI)	*P* value	Multivariate analysis OR (95%CI)	*P* value
Age				
≤ 54	1.00 (reference)			
55–74	0.89 (0.52–1.48)	0.623		
≥ 75	1.52 (0.85–2.72)	0.155		
Sex				
F	1.00 (reference)			
M	1.12 (0.76–1.67)	0.562		
ASA				
1–2	1.00 (reference)			
3–4	1.46 (0.98–2.17)	0.06		
BMI ≥ 30				
< 30 kg/m^2^	1.00 (reference)			
≥ 30 kg/m^2^	0.83 (0.55–1.26)	0.38		
Pre-op CRP ≥ 10mg/L				
< 10 mg/L	1.00 (reference)			
≥ 10 mg/L	1.52 (0.99–2.33)	0.054		
T stage				
T1 and T2	1.00 (reference)			
T3 and T4	1.58 (1.01–2.45)	0.044	-	0.088
Node involvement				
Negative	1.00 (reference)			
Positive	0.83 (0.56–1.25)	0.377		
TNM Stage				
I–II	1.00 (reference)			
III	0.89 (0.59–1.34)	0.571		
IV	0.54 (0.13–2.29)	0.40		
Surgical approach				
Open	1.00 (reference)		1.00 (reference)	
Laparoscopic	0.67 (0.44–1.02)	0.061	-	0.16
Robot-assisted	0.46 (0.24–0.89)	0.017	0.50 (0.26–0.95)	0.036
Year of operation				
2008–2013	1.00 (reference)			
2014–2018	1.3 (0.8- 2.11)	0.293		
2019–2023	0.96 (0.58- 1.59)	0.883		
Type of resection				
HAR	1.00 (reference)			
LAR	1.23 (0.78–1.94)	0.365		
APR	1.26 (0.72–2.22)	0.415		
Others	1.73 (0.88–3.37)	0.110		

### Propensity scores matched model

In order to further investigate differences in perioperative outcomes and inflammatory response profiles between LS and RAS, we undertook a propensity score matched analysis. In the total cohort of 376 and 172 patients who underwent LS and RAS, a total of 334 patients were matched by propensity scores (167 in each group). There was a subsequent improvement in balance in the distribution of propensity scores of the two cohorts after matching (Supplementary Fig. 1a and b). In terms of postoperative inflammatory response, RAS was associated with a lower serum CRP at POD 1 (46 mg/l, IQR 30–71 versus LS 56 mg/l, IQR 39–83, *p* = 0.004) and POD 2 (80 mg/l, IQR 52–131 versus LS 107 mg/l, IQR 72–165, *p* = 0.004) but not POD 3 and 4 (Table 5). Furthermore, RAS was associated with a smaller proportion of patients with a CRP ≥ 150 mg/l at POD 3 (20.9% versus 30.5%, *p* = 0.036).

**Table 5:** Baseline clinical and pathological characteristics and outcomes of propensity score matched patients (McNemar test for categorical outcomes data and Wilcoxon signed-rank tests for continuous outcomes data)

Table 1: All patients	LS	RAS	*p*-value
Total resections *n* = 334	167	167	
High	75 (44.9%)	70 (41.9%)	
Low	57 (34.1%)	61 (36.5%)	
APR	25 (15%)	23 (13.8%)	
Other	10 (6%)	13 (7.8%)	
Year 2008–2013	46 (27.5%)	0	
Year 2013–2018	79 (47.3%)	0	
Year 2019–2023	42 (25.2%)	167 (100%)	
Age ≤ 54	29 (17.4%)	35 (21%)	
Age 55–74	109 (65.3%)	100 (59.9%)	
Age ≥ 75	29 (17.4%)	32 (19.2%)	
Sex Female	69 (41.3%)	70 (41.9%)	
BMI ≥ 30 kg/m2	48 (28.9%)	56 (33.5%)	
ASA ≥ 3	62 (37.1%)	60 (35.9%)	
T1	56 (33.5%)	28 916.8%)	
T2	36 (21.6%)	34 (20.4%)	
T3	72 (43.1%)	88 (52.7%)	
T4	3 (1.8%)	17 (10.2%)	
N0	144 (86.2%)	109 (65.3%)	
N1	16 (9.6%)	40 (24%)	
N2	7 (4.2%)	18 (10.8%)	
M0	167 (100%)	161 (96.4%)	
M1	0	6 (3.6%)	
Preop CRP ≥ 10 mg/L	22 (13.2%)	30 (18%)	
Median POD1 CRP (mg/L)	56 (39–83)	46 (30–71)	0.004
Median POD2 CRP (mg/L)	107 (72–165)	80 (51.5–131)	0.004
Median POD3 CRP (mg/L)	110 (65–170)	85 (53–139)	0.101
Median POD4 CRP (mg/L)	77 (41–142)	68 (36–130)	0.923
POD2 CRP ≥ 150 mg/L	42 (25.1%)	29 (17.7%)	0.119
POD3 CRP ≥ 150 mg/L	51 (30.5%)	34 (20.9%)	0.036
Median length of inpatient stay (days)	7 (5–11)	6 (4–9)	0.056
Any complications within 30 days	78 (46.7%)	58 (34.7%)	0.033
Major complications CD3-5 within 30 days	16 (9.6%)	12 (7.2%)	0.541
Death within 30 days	2 (1.3%)	0	N/A
Post-operative CT imaging within 30 days	49 (29.7%)	36 (21.7%)	0.087
Post-operative transfusion within 30 days	5 (3.1%)	7 (4.2%)	1.00
Post-operative SSI within 30 days	16 910.3%)	14 (8.4%)	0.523
Readmission to hospital within 30 days	17 (10.4%)	12 (7.3%)	0.359
Re-operation within 30 days	11 (6.7%)	8 (4.8%)	0.648
Conversion to open	12 (7.2%)	7 (4.2%)	0.481

In terms of perioperative outcomes, RAS was associated a lower postoperative all-complications rate (34.7% versus 46.7%, *p* = 0.033). There were no significant differences between the RAS and LS matched cohort in terms of length of stay, the development of major complications, need for postoperative CT imaging, SSI, re-admission, re-operation, or conversion to open although the trend favoured the RAS outcomes.

## Discussion

In the present prospective observational study, a practice change in our institution is described, with a transition to robotic-assisted surgery (RAS) for all left-sided and rectal cancers since May 2021. Comparisons are made with surgically resected left-sided and rectal cancers operated on over the preceding 14 years. With the introduction of RAS we have observed a far higher proportion of patients (> 85%) operated on via a minimally invasive approach for left-sided colon and rectal cancer to that seen in the previous 14 years. This transition towards MIS has been positive in terms of perioperative outcomes. In unadjusted analysis, compared to OS, we report improved short-term outcomes for both RAS and LS in terms of reduced rates of complications (both all complications and major complications) reduced surgical site infections, reduced rates of blood transfusion, lower length of stay and reduced requirements for post-op CT imaging as well as lower rates of reoperation and readmission. Furthermore, the magnitude of the postoperative inflammatory response is lower with minimally invasive surgery vs open surgery has been reported [16, 43]_._

In unadjusted analysis, when comparing only LS and RAS approaches, we observed lower rates of conversion with RAS. We observed trends favouring RAS versus LS in terms of complication rates and length of stay which did not achieve significance, likely reflecting the modest size of the RAS grouping (*n* = 172) in this study. We did however observe statistically significant differences in favour of lower postoperative CRP profiles with RAS vs LS on POD 1–3 in addition to the proportion of patients breaching the CRP** ≥ **150mg/L threshold on POD2. Postoperative imaging rate (CT scanning) was lower in the RAS cohort, possibly related to lower CRP values which forms an important component in the decision making for ordering cross sectional imaging in the immediate postoperative period in routine clinical practice.

We also report, using multivariable binary logistic regression and propensity score matching analyses, consistent results favouring lower post-op inflammatory response profiles and complication rates with RAS compared to LS. Therefore, this study provides the strongest evidence to date to support the fact that RAS is associated with a lower postoperative systemic inflammatory response when compared to laparoscopic or open surgery. The underlying mechanism is yet to be fully elucidated however some hypotheses include reduced tissue trauma relating to surgical wounds, ports and abdominal tension [2, 43].

In this study we report higher rates of transfusion following open surgery compared with MIS approaches (OS 18% vs LS 3.6% vs RAS 4.1%). Lower rates of transfusion following minimally invasive surgery have been previously reported [27–29]. Such differences are thought to relate to the magnitude of trauma and blood loss associated with surgical intervention. However, the presence of high grade systemic inflammatory responses also impact haemoglobin levels negatively [30, 31]. Therefore inflammatory burden in the perioperative period may play an additive role in determining an increased requirement for transfusion.

In addition, previous studies have reported presence of systemic inflammatory responses in relation to blood transfusion [32]. There is therefore a possibility higher rates of perioperative transfusion could impact magnitude of postoperative inflammatory profiles, particularly in the open surgery group. Following PSM analysis, there was no difference in the proportion of patients who received a postoperative blood transfusion within 30 days between LS and RAS but importantly the difference in postoperative CRP and complication rates remained. Further work is ultimately required to better define these interactions.

This study adds to the literature on colorectal RAS suggesting the more rapid return-to-function and earlier hospital discharge is partly related to the reduction in the magnitude of the surgical insult measured by stress response. Notably these results are reported in the early phase of our RAS journey and more pronounced differences or benefits may be achieved in the coming years.

Previous literature has demonstrated a reduced SIR in LS surgery when compared with OS [13, 16, 28]. Serum CRP is often used as a marker for not only the extent of the SIR but also as predictive biomarker for complications. The magnitude of SIR may also play a role in the development of postoperative complications. The implications of these results are profound in that minimising the surgical trauma may improve postoperative outcomes and therefore all operative practices (surgical, anaesthetic and nursing practices) should be carried out with a view to minimising the postop SIR to reduce complications. The present study defines the shallower postoperative inflammatory response curves associated with both LS and robot-assisted MIS which appear to be related to improved short-term outcomes.

There have been 5 key large scale multicentre randomised controlled trials comparing perioperative outcomes and safety of LS versus OS, which support widespread use of MIS for rectal cancer: CLASICC [33], COREAN [34], COLOR II [35], ACOSOG Z6051 [36] and ALaCaRT [37] with data collection periods ranging from 1996 to 2014. Overall, these studies confirmed comparable rates on anastomotic leak, complications and both prognostic and oncological outcomes, with the latter study (ALaCaRT) demonstrating fewer CD3 + complications.

The largest colorectal RAS trial, ROLARR [4] in 2017, compared perioperative outcomes in RAS and LS rectal resection and confirmed comparable short-term outcomes for both approaches. Several studies have retrospectively investigated LOS, oncological outcomes and complications in RAS vs either LS or OS [33–42], but there is very little literature examining the SIR in colorectal RAS. A small prospective single centre study compared pro-inflammatory cytokines and prolactin on postoperative days 1 & 3 and found RAS results in a less pronounced SIR [43].

One prospective randomized control trial in Denmark, SIRIRALS [44], aims to evaluate the systemic and peritoneal inflammatory response in RAS compared to LS for elective colonic cancer resections with a primary end point expressed as CRP and IL-6 between postoperative days 1–3 as well as LOS and conversion rate. We anticipate this study should generate key discussion points when compared with our data.

There are numerous strengths to the present study, with the benefit of a large, prospective, well-maintained institutional database of all resections going back to 2008. As can be seen in Fig. 2, the transition to RAS was rapid and case selection only applied within the first 2 months of training. The consistency of the surgical team who performed this surgery in the preceding 14 years is also important and since 2017, the majority of left-sided and all primary rectal cancers have been directed to the surgeons who have subsequently trained in RAS. The unbiased routine sampling of serum CRP in all patients in this cohort is a further strength of this study.

The observation that RAS is associated with a lower magnitude of SIR may be due to multiple factors related to reduce tissue trauma during surgery. Firstly, RAS ports pivot at the level of the fascia avoiding traction on the abdominal wall which is seen with OS and to a lesser degree LS surgery. Secondly, the operator controlled fixed retraction, may limit traumatic regrasping of tissues. Our anaesthetic approach with RAS is relatively consistent with avoidance of spinal anaesthesia in the main. Intravenous lignocaine infusions are not currently used. Importantly, the observed differences in stress response are apparent with no difference in BMI or gender of patients between our approach groupings and when advanced T4 tumours are excluded. Irrespective, RAS is a modality that not only treats the tumour but also the postoperative systemic inflammatory response and given that the magnitude of the postoperative systemic inflammatory and complications are associated with poorer long-term outcomes, it may be that long-term outcomes will also be improved in patients undergoing RAS [12, 20, 45].

The limitations of the study relate to the potential for case selection in the operative groupings. Clearly at the outset of RAS and LS surgery within an institution, there will be a degree of case selection during the training period. The first surgeon to train was an experienced LS/ OS rectal cancer surgeon who had undertaken a RAS fellowship at a high-volume centre and so the period of specific case selection was minimised. When additional surgeons trained, the more technically demanding operations were directed to the more experienced RAS surgeons initially. In Table 1 we do not see any difference in BMI or sex distribution across the surgical approach groupings. Comparable rates of T4 disease in the RAS and LS groups are observed, but increased T4 disease in our OS cohort which reflects the inherent case selection employed in routine clinical practice with patients with T4 disease. Potential case selection has been adjusted for, controlling for potential interacting factors by analysing specific outcomes of interest within multivariate binary logistic regression models and a propensity score matched model. While we have included the most important co-variates which are known to influence a postoperative inflammatory response, there are potential confounders which have not been included in these models and which may benefit from further investigation in future prospective studies such as operative duration, specific detailed patient co-morbidities, and surgical learning curve during the introduction of an RAS approach in this study.

In both unadjusted analyses looking at RAS versus LS specifically, the differences in postoperative SIR and short-term outcomes between the two approaches are present but to a lesser magnitude compared to analyses comparing the three approaches. However, as shown in this study, within our propensity scores matched model, as well as in unadjusted analyses, there were still statistically significant differences between the two groups with RAS associated with a lower post-op SIR. Further within our PSM model, RAS was associated with less postoperative all-complications rates. While this may indeed suggest an equivalence in both approaches in terms of this study’s outcomes, these results need to take into account the modest size of our RAS cohort and the aforementioned potential missed confounders.

In the UK, laparoscopic surgery has been widely available for the past 15–20 years. Taking all colorectal cancer resections together, rates of open or lap-converted surgery have remained between 25 and 50% over the past 10 years according to the National Bowel Cancer Audit [46]. These rates are likely to be higher for rectal cancer surgery. Such results indicate MIS rates in the UK remain variable. The reasons for lack of adoption of MIS will be multifactorial, but in the UK, these undoubtedly relate to technical challenges with the equipment and also the availability of skilled assistance. Many of these challenges are overcome with RAS which across the England and Wales up to the end of 2021 still only accounted for < 10% of colorectal cancer procedure approaches. In our institution, we believe availability of RAS has allowed us to realise high rates of MIS due to these improved technical aspects of surgical procedures.

In summary, the present prospective observational study shows that implementation of RAS has delivered a transition towards a high rate of MIS in our institution for left-sided colonic and rectal cancer surgery. This shift away from open surgery has benefited patients with improved perioperative outcomes. We report RAS is associated with lower postoperative SIR, and lower postoperative complications in comparison to both open and laparoscopic surgery. It remains to be determined whether further optimisation of the operative procedure will minimise the postoperative SIR. Ongoing work will establish to what degree the learning curve, surgeon volume and other patient-related factors impact the perioperative stress response in RAS.

### Supplementary Information

Below is the link to the electronic supplementary material.

Supplementary file1 (JPG 42 KB)—a: Histogram of propensity score distribution in the RAS and LS approach groups before matching

Supplementary file2 (JPG 40 KB)— b: Histogram of propensity score distribution in the RAS and LS approach groups after matching

## References

[1] Wang X, Cao G, Mao W, Lao W, He C (2020). Robot-assisted versus LS surgery for rectal cancer: a systematic review and meta-analysis. J Cancer Res Ther.

[2] Ielpo B, Caruso R, Quijano Y, Duran H, Diaz E, Fabra I, Oliva C, Olivares S, Ferri V, Ceron R, Plaza C, Vicente E (2014). Robotic versus LS rectal resection: is there any real difference? A comparative single center study. Int J Med Robot.

[3] Nozawa H, Watanabe T (2017). Robotic surgery for rectal cancer. Asian J Endosc Surg.

[4] Jayne D, Pigazzi A, Marshall H, Croft J, Corrigan N, Copeland J, Quirke P, West N, Rautio T, Thomassen N, Tilney H, Gudgeon M, Bianchi PP, Edlin R, Hulme C, Brown J (2017). Effect of robotic-assisted vs conventional LS surgery on risk of conversion to OS Lsarotomy among patients undergoing resection for rectal cancer: the ROLARR randomized clinical trial. JAMA.

[5] Lei X, Yang L, Huang Z, Shi H, Zhou Z, Tang C, Li T (2021). No beneficial effect on survival but a decrease in postoperative complications in patients with rectal cancer undergoing robotic surgery: a retrospective cohort study. BMC Surg.

[6] Wang Y, Liu Y, Han G, Yi B, Zhu S (2020). The severity of postoperative complications after robotic versus LS surgery for rectal cancer: a systematic review, meta-analysis and meta-regression. PLoS ONE.

[7] Chang YS, Wang JX, Chang DW (2015). A meta-analysis of robotic versus LS colectomy. J Surg Res.

[8] Park SY, Choi GS, Park JS, Kim HJ, Ryuk JP, Yun SH (2014). Urinary and erectile function in men after total mesorectal excision by LS or robot-assisted methods for the treatment of rectal cancer: a case-matched comparison. World J Surg.

[9] Ahmed J, Cao H, Panteleimonitis S, Khan J, Parvaiz A (2017). Robotic vs LS rectal surgery in high-risk patients. Colorectal Dis.

[10] Mayor N (2022). Past, present and future of surgical robotics. Trends Urol Men’s Health.

[11] Sheetz KH, Claflin J, Dimick JB (2020). Trends in the adoption of robotic surgery for common surgical procedures. JAMA Netw OS.

[12] McSorley ST, Watt DG, Horgan PG, McMillan DC (2016). Postoperative systemic inflammatory response, complication severity, and survival following surgery for colorectal cancer. Ann Surg Oncol.

[13] Platt JJ, Ramanathan ML, Crosbie RA, Anderson JH, McKee RF, Horgan PG, McMillan DC (2012). C-reactive protein as a predictor of postoperative infective complications after curative resection in patients with colorectal cancer. Ann Surg Oncol.

[14] Giaccaglia V, Salvi PF, Antonelli MS, Nigri G, Pirozzi F, Casagranda B, Giacca M, Corcione F, de Manzini N, Balducci G, Ramacciato G (2016). Procalcitonin reveals early dehiscence in colorectal surgery: the PREDICS study. Ann Surg.

[15] McMillan DC (2013). The systemic inflammation-based Glasgow Prognostic Score: a decade of experience in patients with cancer. Cancer Treat Rev.

[16] Watt DG, McSorley ST, Horgan PG, McMillan DC (2015). Enhanced recovery after surgery: which components, if any, impact on the systemic inflammatory response following colorectal surgery?: A systematic review. Medicine (Baltimore).

[17] Clavien PA, Barkun J, de Oliveira ML, Vauthey JN, Dindo D, Schulick RD, de Santibañes E, Pekolj J, Slankamenac K, Bassi C, Graf R, Vonlanthen R, Padbury R, Cameron JL, Makuuchi M (2009). The Clavien-Dindo classification of surgical complications: five-year experience. Ann Surg.

[18] Royal College of Pathologists (n.d.) TNM classification of colorectal tumours (UICC TNM8). https://www.rcpath.org/profession/guidelines/cancer-datasets-and-tissue-pathways.html

[19] McMahon RK, O'Cathail SM, Nair H, Steele CW, Platt JJ, Digby M, McDonald AC, Horgan PG, Roxburgh SD (2023). The neoadjuvant rectal score and a novel magnetic resonance imaging based neoadjuvant rectal score are stage independent predictors of long-term outcome in locally advanced rectal cancer. Colorectal Dis.

[20] Dolan RD, McSorley ST, Horgan PG, Laird B, McMillan DC (2017). The role of the systemic inflammatory response in predicting outcomes in patients with advanced inoperable cancer: systematic review and meta-analysis. Crit Rev Oncol/Hematol.

[21] Køstner AH, Kersten C, Löwenmark T, Ydsten KA, Peltonen R, Isoniemi H, Haglund C, Gunnarsson U, Isaksson B (2016). The prognostic role of systemic inflammation in patients undergoing resection of colorectal liver metastases: C-reactive protein (CRP) is a strong negative prognostic biomarker. J Surg Oncol.

[22] Thomsen M, Kersten C, Sorbye H, Skovlund E, Glimelius B, Pfeiffer P, Johansen JS, Kure EH, Ikdahl T, Tveit KM, Christoffersen T, Guren TK (2016). Interleukin-6 and C-reactive protein as prognostic biomarkers in metastatic colorectal cancer. Oncotarget.

[23] Kersten C, Louhimo J, Ålgars A, Lahdesmaki A, Cvancerova M, Stenstedt K, Haglund C, Gunnarsson U (2013). Increased C-reactive protein implies a poorer stage-specific prognosis in colon cancer. Acta Oncol.

[24] Ellis CT, Maykel JA (2021). Defining anastomotic leak and the clinical relevance of leaks. Clin Colon Rectal Surg.

[25] Cuschieri S (2019). The STROBE guidelines. Saudi J Anaesth.

[26] Fujikawa H, Okugawa Y, Yamamoto A, Imaoka H, Shimura T, Kitajima T, Kawamura M, Yasuda H, Okita Y, Yokoe T, Ohi M, Toiyama Y (2021). Cumulative C-reactive protein in the perioperative period as a novel marker for oncological outcome in patients with colorectal cancer undergoing curative resection. J Anus Rectum Colon.

[27] Trastulli S, Cirocchi R, Listorti C, Cavaliere D, Avenia N, Gullà N, Giustozzi G, Sciannameo F, Noya G, Boselli C (2012). Laparoscopic vs open resection for rectal cancer: a meta-analysis of randomized clinical trials. Colorectal Dis.

[28] Wiklund E, Carlander J, Wagner P, Engdahl M, Chabok A, Nikberg M (2023). Lower need for allogeneic blood transfusion after robotic low anterior resection compared with open low anterior resection: a propensity score-matched analysis. J Robot Surg.

[29] McKay GD, Morgan MJ, Wong SK, Gatenby AH, Fulham SB, Ahmed KW, Toh JW, Hanna M, Hitos K, South Western Sydney Colorectal Tumor Group (2012). Improved short-term outcomes of laparoscopic versus open resection for colon and rectal cancer in an area health service: a multicenter study. Dis Colon Rectum.

[30] Weiss G, Ganz T, Goodnough LT (2019). Anemia of inflammation. Blood.

[31] Marques O, Weiss G, Muckenthaler MU (2022). The role of iron in chronic inflammatory diseases: from mechanisms to treatment options in anemia of inflammation. Blood.

[32] McSorley ST, Tham A, Dolan RD, Steele CW, Ramsingh J, Roxburgh C, Horgan PG, McMillan DC (2020). Perioperative blood transfusion is associated with postoperative systemic inflammatory response and poorer outcomes following surgery for colorectal cancer. Ann Surg Oncol.

[33] Slim K (2005). MRC CLASICC trial. Lancet.

[34] Jeong SY, Park JW, Nam BH, Kim S, Kang SB, Lim SB, Choi HS, Kim DW, Chang HJ, Kim DY, Jung KH, Kim TY, Kang GH, Chie EK, Kim SY, Sohn DK, Kim DH, Kim JS, Lee HS, Kim JH, Oh JH (2014). OS versus LS surgery for mid-rectal or low-rectal cancer after neoadjuvant chemoradiotherapy (COREAN trial): survival outcomes of an OS-label, non-inferiority, randomised controlled trial. Lancet Oncol.

[35] van der Pas MH, Haglind E, Cuesta MA, Fürst A, Lacy AM, Hop WC, Bonjer HJ, COlorectal cancer LS or OS Resection II (COLOR II) Study Group (2013). LS versus OS for rectal cancer (COLOR II): short-term outcomes of a randomised, phase 3 trial. Lancet Oncol.

[36] Fleshman J, Branda M, Sargent DJ, Boller AM, George V, Abbas M, Peters WR, Maun D, Chang G, Herline A, Fichera A, Mutch M, Wexner S, Whiteford M, Marks J, Birnbaum E, Margolin D, Larson D, Marcello P, Posner M, Read T, Monson J, Wren SM, Pisters PW, Nelson H (2015). Effect of LS-assisted resection vs OS resection of stage II or III rectal cancer on pathologic outcomes: the ACOSOG Z6051 Randomized Clinical Trial. JAMA.

[37] Stevenson AR, Solomon MJ, Lumley JW, Hewett P, Clouston AD, Gebski VJ, Davies L, Wilson K, Hague W, Simes J (2015). ALaCaRT Investigators. Effect of LS-assisted resection vs OS resection on pathological outcomes in rectal cancer: the ALaCaRT randomized clinical trial. JAMA.

[38] Fleming CA, Ullah MF, Chang KH, McNamara E, Condon E, Waldron D, Coffey JC, Peirce CB (2021). Propensity score-matched analysis comparing LS to robotic surgery for colorectal cancer shows comparable clinical and oncological outcomes. J Robot Surg.

[39] van Harten MJ, Greenwood EB, Bedrikovetski S, Dudi-Venkata NN, Hunter RA, Kroon HM, Sammour T (2020). Minimally invasive surgery in elderly patients with rectal cancer: an analysis of the bi-national colorectal cancer audit (BCCA). Eur J Surg Oncol.

[40] Fleming CA, Westby D, Ullah MF, Mohan HM, Sehgal R, Bolger JC, O'Leary DP, McNamara E, Korpanty G, El Bassiouni M, Condon E, Coffey JC, Peirce C (2020). A review of clinical and oncological outcomes following the introduction of the first robotic colorectal surgery programme to a university teaching hospital in Ireland using a dual console training platform. J Robot Surg.

[41] Zhu XL, Yan PJ, Yao L, Liu R, Wu DW, Du BB, Yang KH, Guo TK, Yang XF (2019). Comparison of short-term outcomes between robotic-assisted and LS surgery in colorectal cancer. Surg Innov.

[42] Ng KT, Tsia AKV, Chong VYL (2019). Robotic versus conventional LS surgery for colorectal cancer: a systematic review and meta-analysis with trial sequential analysis. World J Surg.

[43] Zawadzki M, Krzystek-Korpacka M, Gamian A, Witkiewicz W (2017). Comparison of inflammatory responses following robotic and OS colorectal surgery: a prospective study. Int J Colorectal Dis.

[44] Cuk P, Pedersen AK, Lambertsen KL, Mogensen CB, Nielsen MF, Helligsø P, Gögenur I, Ellebæk MB (2021). Systemic inflammatory response in robot-assisted and LS surgery for colon cancer (SIRIRALS): study protocol of a randomized controlled trial. BMC Surg.

[45] McSorley ST, Horgan PG, McMillan DC (2016). The impact of the type and severity of postoperative complications on long-term outcomes following surgery for colorectal cancer: a systematic review and meta-analysis. Crit Rev Oncol Hematol.

[46] Healthcare Quality Improvement Partnership (2023) National Bowel Cancer Audit 2022 annual report https://www.nboca.org.uk/content/uploads/2023/01/NBOCA-2022-Final.pdf

