# Supplementary figures and images for: Robotic-assisted surgery for left-sided colon and rectal resections is associated with reduction in the postoperative surgical stress response and improved short-term outcomes: a cohort study
Source: Surg Endosc. 2024 Mar 18;38(5):2577–92. doi: 10.1007/s00464-024-10749-3 (PMC11078791; doi:10.1007/s00464-024-10749-3)

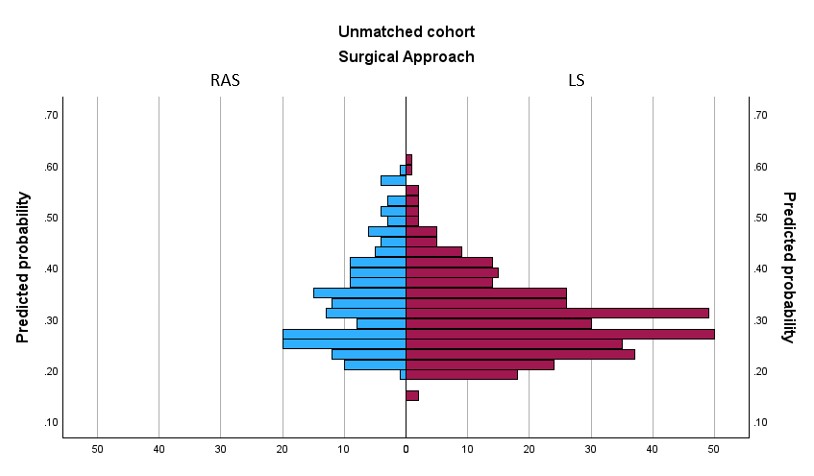

Supplement: Supplementary file 1 — Supplementary file1 (JPG 42 KB)—a: Histogram of propensity score distribution in the RAS and LS approach groups before matching [file 464_2024_10749_MOESM1_ESM.jpg]

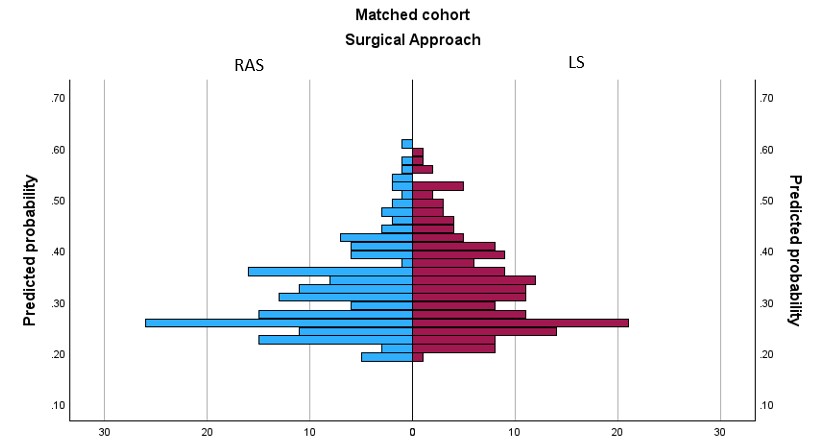

Supplement: Supplementary file 2 — Supplementary file2 (JPG 40 KB)— b: Histogram of propensity score distribution in the RAS and LS approach groups after matching [file 464_2024_10749_MOESM2_ESM.jpg]
